# Ultra-processed foods consumption and subsequent mortality in a cohort of Black breast cancer survivors

**DOI:** 10.1016/j.eclinm.2025.103700

**Published:** 2025-12-17

**Authors:** Tengteng Wang, Elisa V. Bandera, Marley Perlstein, Nur Zeinomar, Karen Pawlish, Coral Omene, Kitaw Demissie, Christine B. Ambrosone, Chi-Chen Hong, Bo Qin

**Affiliations:** a Rutgers Cancer Institute, New Brunswick, NJ, USA; b Robert Wood Johnson Medical School, New Brunswick, NJ, USA; c Rutgers School of Public Health, Piscataway, NJ, USA; d New Jersey State Cancer Registry, Trenton, NJ, USA; e SUNY Downstate School of Public Health, Brooklyn, NY, USA; f Roswell Park Comprehensive Cancer Center, Buffalo, NY, USA

**Keywords:** Ultra-processed foods, Mortality, Black women, Breast cancer survivorship

## Abstract

**Background:**

Epidemiological evidence on the influence of ultra-processed foods (UPFs) on breast cancer prognosis is scarce. No study has examined the UPF-mortality association among Black breast cancer survivors.

**Methods:**

We examined the UPF-mortality relationships among Black women diagnosed with primary breast cancer in New Jersey between 2005 and 2019 (n = 1733), who were participants of the Women's Circle of Health Study and Women's Circle of Health Follow-Up Study. Foods and drinks consumed one year before breast cancer diagnosis were assessed during home interviews by validated food-frequency questionnaires. UPFs were classified according to their degree of processing using the standard NOVA classification system. Death outcomes were ascertained through linkage with New Jersey State Cancer Registry files. Multivariable-adjusted hazard ratios (HRs) and 95% confidence intervals (CIs) for UPF-mortality associations were estimated using Cox and competing risks models, as appropriate.

**Findings:**

After a median of 9.3 years of follow-up since diagnosis, 394 total deaths (206 breast cancer-related) were identified. In the multivariable-adjusted model, compared to those in the lowest tertile (median = 2.6 servings/day), women in the highest tertile (median = 8.1 servings/day) of UPF intake had statistically significantly higher breast cancer-specific mortality (HR = 1.40, 95% CI = 1.00–1.96, P_trend_ = 0.02) and all-cause mortality (HR = 1.36, 95% CI = 1.06–1.74, P_trend_ <0.01). Dose-response analyses revealed a J-shaped association of UPF intake with mortality outcome, with lower risk at under 4 servings/day and higher risk at greater consumption (breast cancer mortality P_for nonlinearity_ = 0.01). These associations were attenuated after additional adjustment for total energy intake.

**Interpretation:**

This large investigation of UPFs and breast cancer prognosis among Black breast cancer survivors suggests that higher consumption of UPFs, which is associated with greater total energy intake, may adversely influence breast cancer prognosis among Black women, a vulnerable population facing the highest risk of breast cancer mortality in the US.

**Funding:**

This work was supported by grants from R01CA185623 (Drs. Bandera, Demissie, and Hong), P30CA072720-5929 (Dr. Bandera), R01CA100598 (Dr. Ambrosone), P01CA151135 (Drs Ambrosone, Palmer, and Olshan), R00CA267557 (Dr. Wang) from the National Cancer Institute, and funding from the American Cancer Society RSG-23-1143513-01-CTPS (Dr. Qin) and the Breast Cancer Research Foundation (Dr. Ambrosone). The New Jersey State Cancer Registry, Cancer Epidemiology Services, New Jersey Department of Health, is funded by the Surveillance, Epidemiology and End Results (SEER) Program of the National Cancer Institute under contract 75N91021D00009, the National Program of Cancer Registries (NPCR), Centers for Disease Control and Prevention under grant NU58DP007117 as well as the State of New Jersey and the Rutgers Cancer Institute.

**Research in context**

**Evidence before this study**

We searched PubMed using the terms ultra-processed food (all fields) and breast cancer mortality or survival (all fields), covering publications from database inception to June 29, 2025. We identified only one study, based on the UK Biobank, that examined the association between pre-diagnostic UPF intake and cancer-related mortality (including deaths from breast cancer), reporting a hazard ratio of 1.22. However, no studies to date have specifically investigated this association among Black women with breast cancer, a medically underserved and vulnerable population disproportionately affected by poor outcomes after diagnosis.

**Added value of this study**

To our knowledge, this is the first investigation of UPF consumption and breast cancer prognosis among Black breast cancer survivors. In this large cohort study, women with the highest level of pre-diagnostic UPF intake had a 36%–40% higher rate of breast cancer-specific and all-cause mortality compared to those with the lowest intake.

**Implications of all the available evidence**

Higher consumption of UPFs may adversely affect breast cancer prognosis among survivors. Further epidemiologic studies in racially and ethnically diverse populations are needed to validate these findings and clarify causality. These results may help inform dietary interventions aimed at reducing mortality risk among Black women with breast cancer.

## Introduction

Breast cancer is a major cause of morbidity and mortality for women in the United States (US),^1^ with mortality rates remaining disproportionately high among Black women, with ∼40% higher mortality for Black women compared to their White counterparts.^2^ Unraveling modifiable factors contributing to the disproportionate burden of poor breast cancer outcomes among Black women following diagnosis remains a critical gap and priority in cancer research.

Accumulating evidence suggests that an unhealthy diet may significantly worsen cancer outcomes following diagnosis.^3^ In particular, ultra-processed foods (UPFs) have recently received major attention as a threat to public health. UPFs are a category of food products that have undergone extensive industrial processing and contain multiple additives, resulting in highly convenient, palatable products with a long shelf life.^4^ They also tend to be highly energy-dense and nutrient-poor. UPFs account for approximately 60% of the daily caloric intake of the US population, a proportion that has increased over the past two decades.^5^ Several large cohort studies conducted in the US and Europe reported positive associations between UPF consumption and mortality from all-cause and cardiovascular disease (CVD)-related mortality in the general population.^6^ Notably, major components of UPFs have also been associated with a higher mortality risk following a breast cancer diagnosis.^7^^,^^8^ These findings provide a strong rationale and support the hypothesis that higher UPF intake may be associated with worse survival outcomes among breast cancer survivors.

Despite plausible mechanisms, epidemiological studies evaluating the impact of UPFs in association with breast cancer survival following breast cancer diagnosis and treatment are extremely scarce. More importantly, no studies have specifically examined the UPF-mortality association among Black women with breast cancer, a medically underserved and vulnerable population disproportionately affected by poor outcomes following diagnosis. To address this crucial knowledge gap, we examined the association of UPF consumption with all-cause and breast cancer-specific mortality among Black breast cancer survivors enrolled in the Women's Circle of Health Study (WCHS) and Women's Circle of Health Follow-Up Study (WCHFS).

## Methods

### Study design

The WCHFS represents one of the largest longitudinal investigations of Black breast cancer survivors to date, and its detailed study design has been described previously.^9^ Briefly, WCHFS was launched in 2013, leveraging the infrastructure of the WCHS (2005–2013), a case-control study focused on investigating risk factors for aggressive breast cancer in Black women.^10^ The WCHFS incorporated Black breast cancer cases from NJ, expanded recruitment using the same methodology, and added active follow-up with repeated annual home visits and phone interviews, as well as blood collection for cases diagnosed after 2013. Mortality outcomes for all breast cancer cases diagnosed from 2005 to 2019 were obtained through data linkage with the New Jersey State Cancer Registry (NJSCR).^9^^,^^11^

Breast cancer case identification was facilitated through rapid case ascertainment across ten counties in New Jersey by NJSCR staff, with cases defined according to ICD-O-3 primary sites C500–C509 and excluding certain histology, such as lymphomas (ICD-O-3 histology codes 9050–9055, 9140, 9590–9993).^9^ Eligible participants were women who self-identified as Black or African American, had a histologically confirmed breast cancer diagnosis, were between 20 and 75 years old at diagnosis, and had no prior history of cancer other than nonmelanoma skin cancer.^9^ The study enrolled approximately 1900 Black women with breast cancer who completed baseline/initial home interviews at approximately 10 months post-diagnosis.^9^ These interviews involved various assessments, including anthropometric and blood pressure measurements, along with comprehensive questionnaires.^9^

### Ethics

The institutional review boards of Rutgers University and Roswell Park Comprehensive Cancer Center approved the study protocols of WCHS and WCHFS (approval number: Pro2017000069). Written informed consents were obtained after participants fully understood the nature of the study, its benefits, risks, and alternatives, and had the consent document fully explained to them in a language they understood. We adhered to the STROBE (Strengthening the Reporting of Observational Studies in Epidemiology) guidelines for reporting cohort studies.

### Exposure (UPFs) assessment

During the initial home interview, participants were asked about their typical dietary intake 1 year before diagnosis. We used either the validated 125-item Fred Hutchinson food frequency questionnaire (FFQ) (n = 484, for women who were diagnosed during 2005–2012) or the validated 85-item modified NCI Block FFQ (n = 1,450, for women diagnosed since 2013).^12^ The Fred Hutchinson FFQ was validated in the Women's Health Initiative Dietary Assessment Study,^13^ and the NCI Block FFQ was validated and modified within the Black Women's Health Study.^12^^,^^14^

Using reported frequency and portion size data in the FFQ, food items were categorized based on their level of processing using the NOVA classification system developed by Monteiro et al.^15^, ^16^, ^17^ NOVA categorizes foods into four groups: unprocessed or minimally processed foods, processed culinary ingredients, processed foods, and UPFs.^15^, ^16^, ^17^ UPFs were further classified into mutually exclusive subgroups (see Supplemental Table S1), including breads/breakfast foods, processed meats, sweet/desserts, sugar- or artificially sweetened beverages, dairy products, mixed dishes, dressings/sauces, and packaged savory snacks.

Preliminary analyses showed that total energy intake and the distribution of major UPF subgroups, such as sweets and processed meats, were consistent across both FFQ administrations. In the current analysis, we excluded participants with missing dietary data (n = 160) or implausible total daily energy intake (i.e., <400 or >6500 kcal/day; n = 20). Comparisons of key demographic and clinical characteristics between participants who completed the FFQ and nonresponders showed no meaningful differences (data not shown).

### Outcome assessment

The primary outcomes of interest were breast cancer-specific mortality and all-cause mortality. Vital status, causes of death, and date of death were determined through linkage with the NJSCR files.^9^ These files are updated annually using various sources, including state death certificates and National Death Index records, hospital discharge files, Medicare and Medicaid databases, and Social Security Administrative Data.^9^ The most recent NJSCR linkage update was completed in November 2024. Participants were censored at the date of death or date of last contact, whichever came first.

### Confounding/covariate assessment

Information on potential confounding factors was collected through in-person baseline home interviews and data linkages.^9^ The questionnaire administered during the home interview collected data on a wide range of factors, including sociodemographic factors, personal and family medical history, reproductive health, medication usage, and lifestyle behaviors.^9^ Neighborhood characteristics at the census tract level were incorporated using NCI's neighborhood socioeconomic status (nSES) Yost index,^18^ based on participants' residence at diagnosis.^19^^,^^20^ Missing values for key covariates were mostly in fewer than five cases and were excluded from the analyses. Missing income was present for 32 participants and was handled using a missing-indicator category. The final analytic sample consisted of 1733 women with primary breast cancer.

During the baseline interview, participants were asked to provide consent for medical record review, with approximately 98% consenting.^9^ Details on breast cancer treatment were abstracted from medical records^9^^,^^11^^,^^19^; if unavailable, NJSCR or self-reported data were used instead.^9^^,^^11^^,^^19^ Prior analysis demonstrated high concordance between self-reported and medical record treatment data (kappa 0.91 for chemotherapy and 0.74 for radiation therapy).^9^^,^^11^^,^^19^

Similarly, information on hypertension and diabetes history was obtained from medical records or, when unavailable, through self-report. Hypertension status was also assessed using measured systolic and diastolic blood pressure during home interviews.^9^^,^^11^^,^^19^ Tumor clinicopathologic features, including American Joint Committee on Cancer (AJCC) stage, tumor grade, estrogen receptor (ER) and progesterone receptor (PR), and human epidermal growth factor receptor 2 (HER2) status, were abstracted from pathology reports and supplemented with data linkage with the NJSCR.^9^

### Statistical analysis

UPF consumption was analyzed both as a continuous variable and categorized into tertiles. Restricted cubic splines with four knots at the 5th, 35th, 65th, and 95th centiles were used to flexibly model the association between UPF consumption and mortality. Multivariable-adjusted Cox proportional hazards regression models were used to examine the association between UPF consumption with both overall and breast cancer-specific mortality.^21^ Deaths from causes other than breast cancer were treated as competing events. The proportional hazards assumption was assessed by visual inspection of log (−log(S)) plots and by testing time-dependent covariate interactions for potential violations.^21^ Competing risks analysis was performed using the Fine-Gray model to estimate the sub-distribution HR (sHR). Potential confounders were selected *a priori* based on existing literature and informed by a directed acyclic graph (DAG) (Supplemental Figure S1).^22^ The program DAGitty was used to identify the final minimally sufficient adjustment set, which included age at diagnosis, educational level, health insurance status, household income, nSES, and marital status assessed at the initial interview. Before regression analysis, multicollinearity among predictors was assessed using Variance Inflation Factors (VIF).

Total energy intake was included as a covariate only in our secondary models. Because energy intake may act as both a confounder (underlying energy requirements or eating patterns of our participants influence both UPF consumption and mortality) and a mediator (if UPF consumption increases total caloric intake, which in turn affects mortality). To account for this complexity, we presented models with and without energy adjustment to illustrate its potential influence on the observed associations. Energy-adjusted UPF consumption was further modeled using two complementary approaches: first, by expressing UPF intake as a percentage of total energy intake; and second, by applying both the nutrient density method and the residual method^23^ as sensitivity analyses. The VIF values for UPF (4.26) and total energy consumption (4.32) indicated moderate correlations but remained below the commonly problematic collinearity threshold of 5. Therefore, we presented both energy-adjusted and unadjusted models for comparison and transparency.

Other sensitivity analyses for fitting additional models based on the DAG-defined model were also performed by adjusting for breast cancer clinical characteristics, as they are strong predictors of mortality outcomes, although they might lie on the causal pathway. We also tested models adjusting for lifestyle and medical factors to assess the robustness of the findings under alternative covariate adjustment strategies.

In secondary analyses, we examined specific UPF components. Stratified analyses were conducted by menopausal status, tumor ER status, AJCC stage, molecular subtype, BMI and waist circumference, and nSES. Effect measure modification on the multiplicative scale was assessed using likelihood ratio tests to compare models with and without interaction terms between UPF intake and each stratifying variable. We also performed mediation analysis^24^ to explore how much of the observed association is mediated by the relationship between UPF and total energy consumption.

### Role of the funding sources

The funding agencies listed in the acknowledgement section were not involved in the design, conduct, analysis, or interpretation of the study.

## Results

**Fig. 1:**
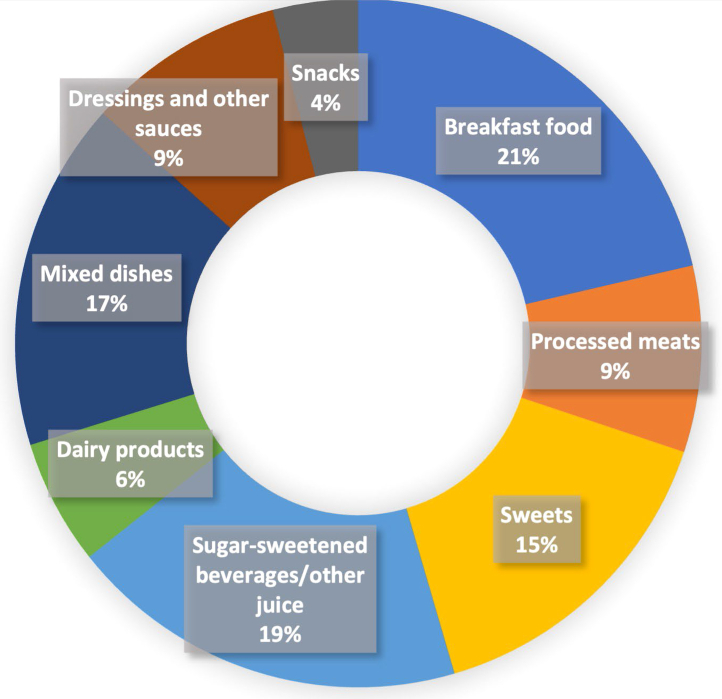
Proportion of individual UPFs subgroup in total UPFs intake.

Among 1733 Black women with breast cancer (Table 1), the mean age at diagnosis was 54.3 years. Women with higher pre-diagnosis UPF consumption were more likely to be diagnosed at a younger age, have lower individual and neighborhood SES, and exhibit higher BMI and waist circumference. They also reported higher alcohol consumption, were more likely to be ever-smokers, had comorbidities, and were more likely to be diagnosed with advanced-stage tumors and to receive chemotherapy. The average intake of total UPFs was 5.47 servings/day. Among the top three major UPF subgroups, breakfast foods accounted for 21% of total UPF consumption, followed by sugar-sweetened beverages and other juices (19%), and mixed dishes (17%) (Fig. 1).

**Table 1:** Characteristics of Black women with breast cancer from the Women's Circle of Health and Women's Circle of Health Follow-Up Study, according to pre-diagnosis (1 year before diagnosis) ultra-processed foods (UPFs) consumption (N = 1733).

Characteristics	Overall (n = 1733)	Tertile 1 (n = 579)	Tertile 2 (n = 576)	Tertile 3 (n = 578)
Age at diagnosis, mean (standard deviation, SD)	54.3 (10.7)	56.4 (10.3)	54.2 (10.2)	52.2 (11.2)
Education, HS grad or less, %	653 (37.7)	208 (35.9)	198 (34.4)	247 (42.7)
Marital status, Single/Never married, %	505 (29.1)	151 (26.1)	156 (27.1)	198 (34.3)
Household income <15,000 ($), %	564 (32.5)	148 (25.6)	178 (30.9)	238 (41.2)
nSES, mean (SD)	9466.6 (904.1)	9564.9 (953.8)	9512.6 (928.5)	9322.3 (806.6)
Health Insurance, Uninsured, %	178 (10.3)	61 (10.5)	64 (11.1)	53 (9.2)
Postmenopausal, %	1059 (61.1)	396 (68.4)	348 (60.4)	315 (54.5)
Ever smoker, %	690 (39.8)	204 (35.2)	209 (36.3)	277 (47.9)
Body mass index, kg/m^2^, mean (SD)	31.9 (7.1)	31.6 (6.9)	31.7 (7.1)	32.6 (7.2)
Waist circumference, cm, mean (SD)	101.6 (15.5)	100.6 (15.3)	100.9 (15.2)	103.3 (16.0)
Hip circumference, cm, mean (SD)	113.7 (13.8)	113.1 (13.5)	113.2 (13.5)	114.7 (14.2)
Body fat percentage, mean (SD)	41.2 (7.5)	41.1 (7.0)	40.8 (7.8)	41.7 (7.6)
Fat mass index, mean (SD)	13.5 (5.0)	13.3 (4.9)	13.3 (5.0)	14.0 (5.1)
Physical activity, not meeting guidelines, %	1094 (63.1)	366 (63.2)	376 (65.3)	352 (60.9)
Total calories intake, kcal, mean (SD)	1794.1 (884.7)	1113.4 (375.4)	1621.8 (413.1)	2647.8 (901.9)
Alcohol consumption, drinks/week, mean (SD)	1.43 (4.23)	1.10 (3.40)	1.38 (3.97)	1.82 (5.10)
Family history of breast cancer, %	303 (17.5)	92 (15.9)	106 (18.4)	105 (18.2)
History of hypertension, %	985 (56.8)	321 (55.4)	325 (56.4)	339 (58.7)
History of type 2 diabetes, %	380 (21.9)	124 (21.4)	124 (21.5)	132 (22.8)
Stage III and IV breast cancer, %	225 (13.0)	64 (11.1)	78 (13.5)	83 (14.4)
Estrogen receptor (ER) negative breast cancer, %	474 (27.4)	159 (27.5)	154 (26.7)	161 (27.9)
Surgery, %	1677 (96.8)	565 (97.6)	557 (96.7)	555 (96.0)
Chemotherapy, %	948 (54.7)	285 (49.2)	311 (54.0)	352 (60.9)
Radiation therapy, %	1170 (67.5)	407 (70.3)	383 (66.5)	380 (65.7)
Endocrine therapy, %	1118 (64.5)	374 (64.6)	368 (63.9)	376 (65.1)
Total UPFs consumption, servings/day, mean (SD)	5.47 (3.34)	2.56 (0.77)	4.69 (0.65)	9.15 (3.13)
Subgroups of UPFs consumption, mean (SD)				
Breakfast food, servings/day	1.03 (0.74)	0.65 (0.43)	1.03 (0.58)	1.41 (0.90)
Processed meats, servings/day	0.49 (0.60)	0.21 (0.26)	0.43 (0.39)	0.84 (0.81)
Sweets, servings/day	0.91 (1.06)	0.34 (0.36)	0.71 (0.56)	1.67 (1.41)
Sugar-sweetened beverage/other juice, servings/day	1.17 (1.47)	0.38 (0.45)	0.89 (0.77)	2.23 (1.96)
Dairy products, servings/day	0.27 (0.35)	0.19 (0.26)	0.28 (0.33)	0.35 (0.43)
Mixed dishes, servings/day	0.92 (0.87)	0.40 (0.30)	0.76 (0.50)	1.58 (1.08)
Dressings and other sauces, servings/day	0.45 (0.46)	0.28 (0.30)	0.43 (0.38)	0.65 (0.58)
Snacks, servings/day	0.23 (0.43)	0.11 (0.21)	0.16 (0.28)	0.43 (0.61)

**Fig. 2:**
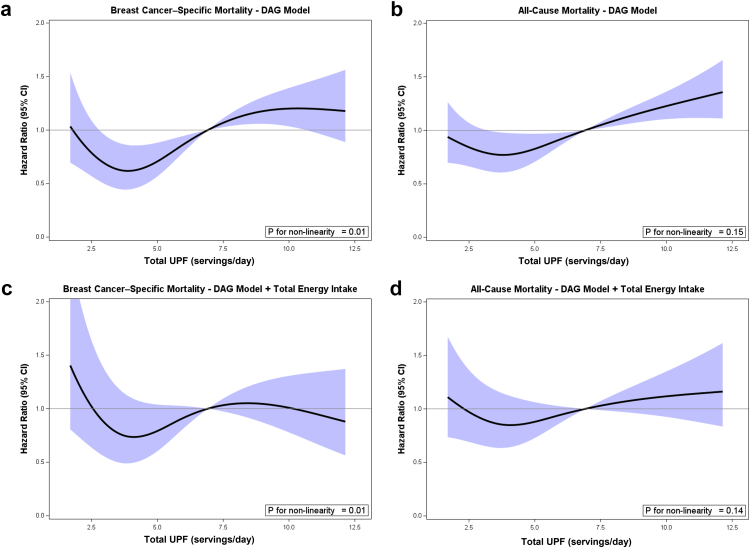
Association of UPFs consumption (servings/day) with breast cancer and all-cause mortality in WCHFS. a: UPFs and breast cancer-specific mortality (DAG model); b: UPFs consumption and all-cause mortality (DAG model); c: UPFs and breast cancer-specific mortality (DAG model + total energy intake). b: UPFs consumption and all-cause mortality (DAG model + total energy intake). Hazard ratios are indicated by solid lines and 95% CIs by shaded areas, with knots placed at 5th, 35th, 65th, and 95th centiles of UPFs distribution.

After a median follow-up of 9.3 years since diagnosis, 394 total deaths were identified, including 206 breast cancer-related deaths. In Fig. 2, we used restricted cubic splines to model and visualize the associations of UPF consumption with two mortality outcomes, with the relationships generally following a J-shaped pattern. A significant non-linear association was observed between total UPF intake and breast cancer-specific mortality (p_for non-linearity_ = 0.01). At lower intakes of UPF, the HR for breast cancer-specific mortality declined, reaching a nadir around 4 servings/day (HR = 0.62; 95% CI: 0.45–0.87). Beyond this point, the HR began to rise and exceeded unity at approximately 7 servings/day, indicating a higher mortality risk at greater levels of UPF consumption. The HR reached 1.20 (95% CI: 1.02–1.42) at the upper range of intake (∼12 servings/day). The association between total UPF intake and all-cause mortality followed a more modest J-shaped pattern, with a roughly linear relationship, with p_for non-linearity_ = 0.15. After further adjustment for total energy intake, the J-shaped association between UPF intake and both mortality outcomes persisted but was attenuated, with wider confidence intervals observed at the extreme ends. Similar attenuation was also observed by modeling energy-adjusted UPF consumption (Supplemental Table S5) using different methods. The attenuation of associations after energy adjustment may indicate that energy intake plays a dual role, acting as both a confounder and a potential mediator, as causal mediation analysis indicated that the proportion mediated was approximately 59% (p = 0.28).

When UPF intake was modeled categorically, higher UPF consumption was significantly associated with higher breast cancer-specific mortality in the DAG-defined model (Table 2, Tertile 3 vs. Tertile 1: HR = 1.40, 95% CI = 1.00–1.96, P_trend_ = 0.02). Similarly, total UPF intake was positively associated with all-cause mortality (HR = 1.36, 95% CI = 1.06–1.74, P_trend_ < 0.01). The association remained statistically significant after adjusting for other lifestyle factors, comorbidities, and menopausal status (Supplemental Table S2) and stratifying by follow-up time (Supplemental Table S3). However, it became attenuated again after adjusting for total energy intake (Table 2) and clinical factors (Supplemental Table S2), as expected.

**Table 2:** Association of pre-diagnosis UPFs consumption with breast cancer specific-mortality and all-cause mortality among Black women with breast cancer from the Women's Circle of Health and Women's Circle of Health Follow-Up Study (n = 1733).

	UPFs consumption by tertiles			P for trend
	Tertile 1 (n = 579)	Tertile 2 (n = 576)	Tertile 3 (n = 578)	
Total UPFs consumption, median (IQR), servings/d	2.61 [2.0,3.2]	4.62 [4.1,5.2]	8.09 [6.9,10.5]	
Breast cancer-specific mortality				
No. of events (n = 206)	62	53	91	
HR (95% CI)^a^ directed acyclic graph (DAG) defined model	Reference (Ref)	0.82 (0.57, 1.19)	1.40 (1.00, 1.96)	0.02
HR (95% CI)^b^ DAG defined model + total energy intake	Ref	0.79 (0.54, 1.16)	1.23 (0.77, 1.97)	0.13
All-cause mortality				
No. of events (n = 394)	126	113	155	
HR (95% CI)^a^ DAG defined model	Ref	0.93 (0.72, 1.19)	1.36 (1.06, 1.74)	<0.01
HR (95% CI)^b^ DAG defined model + total energy intake	Ref	0.86 (0.66, 1.11)	1.06 (0.75, 1.51)	0.27

a DAG defined minimal sufficient adjustment set: age at diagnosis (continuous, years), baseline educational level (≤ high school graduate, some college, ≥ college graduate), health insurance status (Private, Medicare/Medicaid, Uninsured, Unknown), household income (<$15,000, $15,000–$29,999, ≥$30,000, Unknown), nSES (continuous), and marital status (married/living as married, widow/divorced/separated, single/never married).

b Adjustment set: age at diagnosis (continuous, years), baseline educational level (≤ high school graduate, some college, ≥ college graduate), health insurance status (Private, Medicare/Medicaid, Uninsured, Unknown), household income (<$15,000, $15,000–$29,999, ≥$30,000, Unknown), nSES (continuous), marital status (married/living as married, widow/divorced/separated, single/never married), and total energy intake (continuous).

**Fig. 3:**
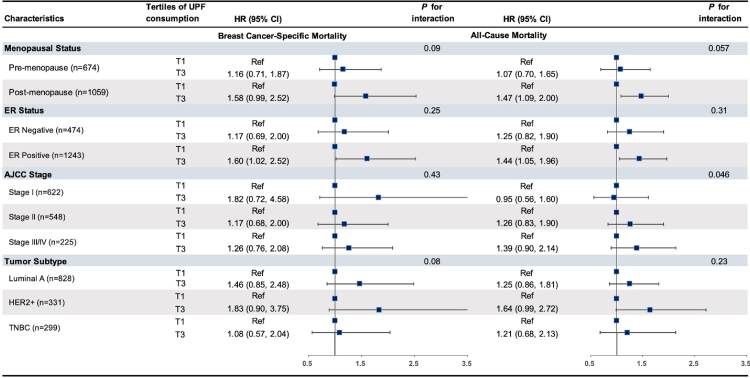
Association of pre-diagnosis UPFs consumption with mortality among Black women with breast cancer in subgroups defined by breast cancer clinical factors, the Women's Circle of Health and Women's Circle of Health Follow-Up Study (n = 1733).

No statistically significant effect modification was observed for the association between UPF consumption and mortality outcomes by menopausal status at diagnosis, tumor ER status, stage, or molecular subtype (p-interaction ≥0.05 and 95%CI overlap across subgroups) (Fig. 3). However, the adverse associations of UPFs on both breast cancer-specific and overall mortality were more pronounced among postmenopausal women and those with ER-positive and/or HER2-positive tumors. These associations were also stronger among participants living in socioeconomically disadvantaged neighborhoods (nSES score < median) (Supplemental Figure S2).

Among the eight UPF subgroups (Supplemental Table S4), higher processed meat consumption was associated with a higher risk of all-cause mortality (HR = 1.34, 95% CI = 1.05–1.73, P_trend_ < 0.01). A similar association was observed for higher intake of mixed dishes (HR = 1.36, 95% CI = 1.06–1.74, P_trend_ = 0.01). Notably, except dairy products, higher consumption of the remaining seven UPF subgroups, including savory snacks, sweets/desserts, and breakfast foods, was generally associated with higher risk of breast cancer-specific mortality, although these associations did not reach statistical significance.

## Discussion

In this large population-based cohort study to investigate the association between UPF consumption and mortality among Black breast cancer survivors, we observed J-shaped associations between UPF and two mortality outcomes, with HRs increasing beyond 4 servings per day and a significant non-linear relationship for breast cancer-specific mortality. When UPF intake was modeled categorically, higher intake (median 8.1 servings per day) was associated with a 40% higher risk of breast cancer-specific mortality and a 36% higher risk of all-cause mortality, compared to lower UPF intake (median 2.3 servings per day). These associations were attenuated after additional adjustment for total energy intake.

Emerging evidence suggests that higher consumption of UPFs is associated with an increased risk of developing breast cancer.^25^ However, epidemiological studies examining the impact of UPF intake on breast cancer survival among cancer survivors remain scarce. To date, only three studies have specifically evaluated the relationship between UPF intake in relation to cancer-specific mortality among survivors.^26^, ^27^, ^28^ Using data from the UK Biobank, Zhao et al. found that a 10% increase in the pre-diagnosis UPFs intake was associated with a 22% higher risk of cancer-related mortality, including deaths from breast cancer, colorectal cancer (CRC), and other malignancies.^26^ Pu et al. observed a similar positive association for CRC and prostate cancer mortality in the PLCO Cancer Screening Trial.^28^ In contrast, Hang et al. did not observe any association between UPF intake and CRC-specific mortality among CRC survivors in the Nurses' Health Study and Health Professionals’ Follow-up Study.^27^ Findings from studies in general healthy populations and a recently published landmark umbrella review of epidemiological meta-analyses^6^ do not support a positive UPF-cancer mortality association.

In contrast to prior findings, the present study observed a 40% increased risk of breast cancer-specific mortality associated with higher UPF consumption. In addition, we observed a J-shaped association between UPF intake and breast cancer-specific mortality, suggesting a complex relationship in which moderate consumption may not have adverse effects, whereas high UPF intake is associated with an increased risk of mortality. Several factors may explain the observed discrepancies. First, unlike most prior studies that focused on the general healthy population, this study specifically examined the UPF-mortality associations in Black women after breast cancer diagnosis and treatment. Second, UPF intake was assessed within one year of cancer diagnosis, capturing exposure more proximally relevant to survival outcomes, whereas the time frame for other cohorts tended to be several years before cancer diagnosis, potentially introducing greater exposure misclassification. Additionally, most prior studies did not report breast cancer-specific mortality, making direct comparisons challenging.

Compelling evidence indicates an adverse effect of UPF intake on overall mortality in the general population^6^^,^^29^; however, its impact on all-cause mortality among Black breast cancer survivors remains unexplored. We found that high UPF consumption is associated with increased overall mortality in our population. These findings likely reflect both breast cancer-related deaths and other causes of death, such as cardiometabolic diseases. Taken together, these findings underscore the potential importance of lowering UPF intake to prevent cardiometabolic diseases and non-cancer deaths.

Consistent with other studies, we observed a strong positive correlation between UPF consumption and total energy intake, likely due to their high energy density. Excessive energy intake contributes further to weight gain and obesity, which are well-established risk factors for various chronic diseases and increased mortality.^6^^,^^29^ In the present study, higher consumption of UPFs was initially associated with an increased risk of mortality. However, this association was no longer statistically significant after adjustment for total energy intake. This attenuation suggests that the observed positive associations between UPFs and mortality may, at least in part, reflect the higher caloric intake associated with these foods rather than an independent effect of UPFs per se. Alternatively, this finding could indicate that part of the detrimental effect of UPFs on mortality operates through increased energy intake, suggesting a potential mediating pathway. Our mediation analysis shows 59% of the effect may be mediated by total energy intake, although the non-significance and wide 95% CI suggest substantial uncertainty around this estimate. Future larger studies are needed to better quantify the direct and indirect effects of UPF on mortality. Taken together, from the point of view of public health, our findings suggest that high UPF consumption may reduce survival among individuals with breast cancer, either through its contribution to excess caloric intake or via direct pathways.

We also observed significant variation in prognostic associations across UPF subgroups, likely driven by differences in their biological effects. Increased consumption of processed meat and mixed dishes was associated with higher overall mortality risk. These foods, often high in unhealthy fats, refined sugars, and low in fiber, contain dietary components linked to poor prognosis in breast cancer patients.^8^ Processed meats, in particular, contain additives and preservatives that may disrupt metabolic processes and promote systemic inflammation, both of which may contribute to cancer progression.^7^^,^^8^ Additionally, food processing can generate toxic compounds,^30^ and the long shelf life of UPFs increases exposure to contaminants from packaging, such as phthalates and microplastics, some with potential carcinogenic effects.^30^

Consistent with our findings, previous research suggests that the adverse impact of UPFs may be more pronounced in specific subgroups, including women with ER-positive tumors^25^ and individuals with lower socioeconomic status who face limited access to healthier, whole food options.^31^ Future larger investigations are needed to elucidate the potential heterogeneity across molecular subtypes and to identify upstream social determinants, including food environment and access, that contribute to high UPF consumption among Black breast cancer survivors.

Our study focuses on Black breast cancer survivors, a population underrepresented in nutritional epidemiology and cancer survivorship research. Socioeconomic and environmental factors, including neighborhood food access, affordability of minimally processed foods, and cultural dietary practices, may shape UPF consumption patterns and modify its association with mortality outcomes. While the biological mechanisms linking UPF to mortality are expected to be similar across racial groups, contextual differences such as chronic stress, comorbidity burden, and disparities in healthcare access may influence the observed magnitude of the association in other populations. Therefore, our findings should be interpreted primarily within the context of Black women in 10 counties in NJ, with the potential limited generalizability of our findings to other races/ethnicities and other regions.^9^ Future research in more racially diverse cohorts could help determine whether the observed associations differ by race or SES status and clarify the broader public health relevance of UPF intake across populations.

This study also has several limitations. First, there is potential for misclassification of UPF intake, as classification relied on FFQ data and the NOVA system, which may not capture the full range or true degree of processing. Certain foods, such as pizza, may be categorized as UPF (NOVA 4) in our study based on their most common industrially produced forms, although they could be classified as processed (NOVA 3) if prepared in artisanal settings or homemade. This potential misclassification may have led to some attenuation of the observed associations. Furthermore, the ongoing debate regarding the conceptual boundaries and practical application of the NOVA framework underscores the need for continued refinement and validation of UPF classification in diverse dietary contexts. We acknowledge that self-reported data may be subject to recall bias and measurement error. Women might also have underreporting of unhealthy foods due to social desirability bias. However, given the prospective study design, as previously mentioned, such misclassification is likely non-differential and may bias association estimates toward the null. Moreover, statistical power was limited for evaluating the association between UPF intake and breast cancer-specific mortality by tumor subtypes and for any mediation analysis; therefore, these findings should be interpreted with caution. Similarly, we were unable to conduct more granular analyses of other causes of death (e.g., CVD death) due to limited power.

The strengths of this study include leveraging data from a large, well-characterized, and prospective cohort of Black breast cancer survivors, with a population-based design that enhances both internal and external validity. Additionally, dietary exposure was assessed during interviews conducted within a similar timeframe, ∼10 months post-diagnosis, across all participants, making dietary data collection more systematic and reducing measurement error due to differences in time since diagnosis. Mortality outcomes were ascertained through the NJSCR, which provides high-quality cancer mortality follow-up information. Given that dietary assessment was independent of subsequent mortality outcomes ascertainment, any misclassification of UPF consumption would likely be non-differential, which tends to bias results toward the null.

In sum, in a population-based cohort of Black women diagnosed with first primary breast cancer and followed for a median of over nine years, higher UPF consumption was associated with both higher all-cause and breast cancer-specific mortality. These findings offer valuable insights into the role of nutrition during cancer survivorship, emphasizing the importance of considering both the nutritional quality of foods and the potential adverse impact of food processing. Our findings contribute to the expanding body of knowledge regarding the role of UPFs in cancer outcomes and add to evidence-based dietary recommendations for cancer survivors and their caregivers. These insights can also inform the development of targeted dietary interventions aiming to help mitigate the adverse cancer outcomes, particularly among Black women, a historically underserved population disproportionately affected by poor breast cancer survival.

## Contributorst

TW, BQ, and EVB contributed to study conception and design, with development of UPF variables and statistical analysis led by TW and MP. TW, BQ, and MP accessed and verified the underlying data. MP and TW carried out primary data analysis. EVB and QB supervised the project. TW wrote the first draft of the manuscript. All authors contributed to critical revision and editing of the manuscript and have approved the final version. TW, BQ, and EVB were responsible for the decision to submit the manuscript. The corresponding author attests that all listed authors meet authorship criteria and that no others meeting the criteria have been omitted. TW, BQ, and EVB had full access to all of the data in the study and take responsibility for the integrity of the data and the accuracy of the data analysis.

## Data sharing statement

The de-identified data underlying this article can be shared upon approval of a data request form by the Women's Circle of Health Follow-up Study Scientific Committee and with appropriate human subject approval and data transfer agreements. The data are not publicly available because they contain protected health information that could compromise the privacy of research participants. Please contact the corresponding author for more information.

## Declaration of interest

Dr. Bandera is supported by the Rutgers Unilever Endowed Chair in Nutrition and Cancer Prevention and served on a Pfizer Inc. Advisory Board to enhance minority participation in clinical trials, both of which are unrelated to this work. All other authors did not report any competing interests related to the research reported in this article.
