# Supplementary material for: Ultra-processed foods consumption and subsequent mortality in a cohort of Black breast cancer survivors
Source: eClinicalMedicine. 2025 Dec 17;91:103700. doi: 10.1016/j.eclinm.2025.103700 (PMC12770944; doi:10.1016/j.eclinm.2025.103700)
Supplement: Supplementary Table and Figure [file mmc1.docx]

**Supplemental Materials**

**Supplemental Table 1**. Detailed list of the food items classified as UPFs across the two types of FFQ

| **UPF subgroups** | **Fred Hutchinson FFQ (n=473)**  **2005 -2013** | **NCI Block FFQ (n=1,301)**  **after 2013** |
| --- | --- | --- |
| ***Breakfast food*** | Cereal, white bread, dark bread, corn bread | Cereal, white bread, dark bread |
| ***Processed meats*** | Sausage, hot dogs, lunch meats | Sausage, hot dogs, lunch meats, bacon |
| ***Sweets*** | Frozen yogurt, ice cream, pudding, pastries, candy | Frozen yogurt, ice cream, pastries, candy |
| ***Sugar-sweetened beverage/other juice*** | Fruit drinks, meal replacement drinks, regular and diet sodas, hard liquor | Fruit drinks, regular and diet sodas, iced tea, hard liquor |
| ***Dairy products*** | Flavored/sugar-sweetened yogurt (non-frozen), cheese sauces | Flavored/sugar-sweetened yogurt (non-frozen), cheese spread |
| ***Mixed dishes*** | Pastas (with sauce), pizza, burritos, potato/macaroni salads, Asian rice/noodles, fried chicken, fried fish, fried potatoes | Pastas (with sauce), pizza, burritos, potpies, fried chicken, fried fish, fried potatoes |
| ***Dressings and other sources*** | Mayonnaise and other dressing | Mayonnaise and other dressing |
| ***Snacks*** | Chips, pretzels, popcorn, crackers | Snacks (corn chips, popcorn) |

| **Supplemental Table 2.** Association of pre-diagnosis UPFs consumption with mortality among Black women with breast cancer by different adjustment sets, the Women’s Circle of Health and Women’s Circle of Health Follow-Up Study (n=1,733) | | | | | | |
| --- | --- | --- | --- | --- | --- | --- |
|  | **UPFs Intake** | | | |  | |
|  | Tertile1  (n=579) | Tertile 2  (n=576) | Tertile3  (n=578) |  |  | *P* for trend |
| Total UPF Intake, median (IQR), servings/d | 2.61 [2.0,3.2] | 4.62 [4.1,5.2] | 8.09 [6.9,10.5] |  |  |  |
| **Breast cancer-specific mortality** |  |  |  |  |  |  |
| No. of events (n=206) | 62 | 53 | 91 |  |  |  |
| Model 1, DAG + total calories intake, HR (95% CI)^a^ | Ref | 0.79 (0.54, 1.16) | 1.23 (0.77, 1.97) |  |  | 0.13 |
| Model 2, DAG + lifestyle + comorbidities, HR (95% CI) ^b^ | Ref | 0.86 (0.59, 1.24) | 1.44 (1.03, 2.01) |  |  | 0.02 |
| Model 3, DAG + clinical factors, HR (95% CI)^c^ | Ref | 0.77 (0.53, 1.12) | 1.24 (0.88, 1.74) |  |  | 0.10 |
| Model 4, DAG + lifestyle + comorbidities + clinical factors, HR (95% CI)^d^ | Ref | 0.84 (0.58, 1.23) | 1.34 (0.94, 1.90) |  |  | 0.05 |
|  |  |  |  |  |  |  |
| **All-cause mortality** |  |  |  |  |  |  |
| No. of events (n=394) | 126 | 113 | 155 |  |  |  |
| Model 1, DAG + total calories intake, HR (95% CI)^a^ | Ref | 0.86 (0.66, 1.11) | 1.06 (0.75, 1.51) |  |  | 0.27 |
| Model 2, DAG + lifestyle + comorbidities, HR (95% CI) ^b^ | Ref | 0.94 (0.73, 1.20) | 1.33 (1.04, 1.71) |  |  | 0.01 |
| Model 3, DAG + clinical factors, HR (95% CI)^c^ | Ref | 0.88 (0.68, 1.14) | 1.24 (0.96, 1.60) |  |  | 0.05 |
| Model 4, DAG + lifestyle + comorbidities + clinical factors, HR (95% CI)^d^ | Ref | 0.90 (0.70, 1.16) | 1.24 (0.96, 1.60) |  |  | 0.07 |
|  |  |  |  |  |  |  |
| **DAG defined minimal sufficient adjustment set**: age at diagnosis (continuous, years) , baseline educational level (≤ high school graduate, some college, ≥ college graduate), health insurance status (Private, Medicare/Medicaid, Uninsured, Unknown), household income (<$15,000, $15,000-$29,999, ≥$30,000, Unknown), nSES (continuous), and marital status (married/living as married, widow/divorced/separated, single/never married)  ^a^ DAG defined model + further adjusted for total calories intake, kcal (continuous)  ^b^ DAG defined model + further adjusted for baseline cigarette smoking (never smoker, former smoker, current smoker), alcohol consumption (continuous, g/day), BMI (continuous kg/m^2^), physical activity (continuous, METs hour/week), comorbidities (diabetes and hypertension), and menopausal status (pre-menopause, post-menopause)  ^c^ DAG defined model + further adjusted for tumor stage (0, I, II, III/IV, Unknown), tumor molecular subtypes (Luminal A, HER2+, TNBC, Unknown), and chemotherapy (yes, no)  ^d^ DAG defined model + further adjusted for baseline cigarette smoking, alcohol consumption, BMI, physical activity, comorbidities (diabetes and hypertension), and menopausal status, tumor stage, tumor molecular subtypes, and chemotherapy | | | | | | |

|  | \| **Supplemental Table 3.** Association of pre-diagnosis UPFs consumption with breast cancer specific-mortality and all-cause mortality, by follow-up years, among Black women with breast cancer from the Women’s Circle of Health and Women’s Circle of Health Follow-Up Study (n=1,733) \| \| \| \| \| \| \| \| --- \| --- \| --- \| --- \| --- \| --- \| --- \| \|  \| **UPFs Consumption by tertiles** \| \| \| \|  \| \| \| Tertile1  (n=579) \| Tertile 2  (n=576) \| Tertile3  (n=578) \|  \|  \| *P* for trend \| \| Total UPFs consumption, median (IQR), servings/d \| 2.61 [2.0,3.2] \| 4.62 [4.1,5.2] \| 8.09 [6.9,10.5] \|  \|  \|  \| \| **Breast cancer-specific mortality** \|  \|  \|  \|  \|  \|  \| \| ***5-year survival*** \|  \|  \|  \|  \|  \|  \| \| No. of events (n=140) \| 42 \| 34 \| 64 \|  \|  \|  \| \| HR (95% CI)^a^ DAG defined model \| Ref \| 0.79 (0.50, 1.26) \| 1.47 (0.98, 2.20) \|  \|  \| 0.03 \| \| HR (95% CI)^b^ DAG defined model + total energy intake \| Ref \| 0.80 (0.50, 1.28) \| 1.48 (0.84, 2.59) \|  \|  \| 0.12 \| \| ***10-year survival*** \|  \|  \|  \|  \|  \|  \| \| No. of events (n=194) \| 58 \| 50 \| 86 \|  \|  \|  \| \| HR (95% CI)^a^ DAG defined model \| Ref \| 0.82 (0.57, 1.21) \| 1.41 (1.00, 2.00) \|  \|  \| 0.02 \| \| HR (95% CI)^b^ DAG defined model + total energy intake \| Ref \| 0.81 (0.55, 1.20) \| 1.31 (0.81, 2.12) \|  \|  \| 0.19 \| \| **All-cause mortality** \|  \|  \|  \|  \|  \|  \| \| ***5-year survival*** \|  \|  \|  \|  \|  \|  \| \| No. of events (n=195) \| 58 \| 46 \| 91 \|  \|  \|  \| \| HR (95% CI)^a^ DAG defined model \| Ref \| 0.78 (0.53, 1.16) \| 1.56 (1.10, 2.21) \|  \|  \| <0.01 \| \| HR (95% CI)^b^ DAG defined model + total energy intake \| Ref \| 0.75 (0.50, 1.13) \| 1.38 (0.86, 2.21) \|  \|  \| 0.11 \| \| ***10-year survival*** \|  \|  \|  \|  \|  \|  \| \| No. of events (n=332) \| 101 \| 88 \| 143 \|  \|  \|  \| \| HR (95% CI)^a^ DAG defined model \| Ref \| 0.87 (0.65, 1.15) \| 1.49 (1.14, 1.95) \|  \|  \| <0.01 \| \| HR (95% CI)^b^ DAG defined model + total energy intake \| Ref \| 0.80 (0.60, 1.08) \| 1.18 (0.81, 1.71) \|  \|  \| 0.26 \| \|  \|  \|  \|  \|  \|  \|  \| \| ^a^ DAG defined minimal sufficient adjustment set: age at diagnosis (continuous, years), baseline educational level (≤ high school graduate, some college, ≥ college graduate), health insurance status (Private, Medicare/Medicaid, Uninsured, Unknown), household income (<$15,000, $15,000-$29,999, ≥$30,000, Unknown), nSES (continuous), and marital status (married/living as married, widow/divorced/separated, single/never married)  ^b^ Adjustment set: age at diagnosis (continuous, years), baseline educational level (≤ high school graduate, some college, ≥ college graduate), health insurance status (Private, Medicare/Medicaid, Uninsured, Unknown), household income (<$15,000, $15,000-$29,999, ≥$30,000, Unknown), nSES (continuous), marital status (married/living as married, widow/divorced/separated, single/never married), and total energy intake (continuous) \| \| \| \| \| \| \|   **Supplemental Table 4.**  Association of baseline UPFs consumption (total and by UPFs subgroups) with mortality among Black women with breast cancer from the Women’s Circle of Health and Women’s Circle of Health Follow-Up Study (n=1,733) | | | | | | |
| --- | --- | --- | --- | --- | --- | --- | --- | --- | --- | --- | --- | --- | --- | --- | --- | --- | --- | --- | --- | --- | --- | --- | --- | --- | --- | --- | --- | --- | --- | --- | --- | --- | --- | --- | --- | --- | --- | --- | --- | --- | --- | --- | --- | --- | --- | --- | --- | --- | --- | --- | --- | --- | --- | --- | --- | --- | --- | --- | --- | --- | --- | --- | --- | --- | --- | --- | --- | --- | --- | --- | --- | --- | --- | --- | --- | --- | --- | --- | --- | --- | --- | --- | --- | --- | --- | --- | --- | --- | --- | --- | --- | --- | --- | --- | --- | --- | --- | --- | --- | --- | --- | --- | --- | --- | --- | --- | --- | --- | --- | --- | --- | --- | --- | --- | --- | --- | --- | --- | --- | --- | --- | --- | --- | --- | --- | --- | --- | --- | --- | --- | --- | --- | --- | --- | --- | --- | --- | --- | --- | --- | --- | --- | --- | --- | --- | --- | --- | --- | --- | --- | --- | --- | --- | --- | --- | --- | --- | --- | --- | --- | --- | --- | --- | --- | --- | --- | --- | --- | --- | --- | --- | --- | --- | --- |
|  | | **UPFs Intake** | | | |  |  |
|  |  | Tertile1 | Tertile 2 | Tertile3 |  |  | *P* for trend |
| **Breast cancer-specific mortality** | |  |  |  |  |  |  |
| Total UPF | | Ref | 0.82 (0.57, 1.19) | 1.40 (1.00, 1.96) |  |  | 0.02 |
| Breakfast Foods | | Ref | 1.13 (0.80, 1.59) | 1.17 (0.83, 1.63) |  |  | 0.37 |
| Processed Meats | | Ref | 0.90 (0.63, 1.28) | 1.16 (0.82, 1.64) |  |  | 0.26 |
| Sweets | | Ref | 0.97 (0.68, 1.38) | 1.21 (0.87, 1.70) |  |  | 0.19 |
| Sugar-Sweetened Beverages | | Ref | 1.21 (0.86, 1.69) | 1.14 (0.81, 1.63) |  |  | 0.58 |
| Dairy | | Ref | 0.92 (0.66, 1.28) | 1.01 (0.72, 1.41) |  |  | 0.88 |
| Mixed Dishes | | Ref | 1.07 (0.75, 1.52) | 1.15 (0.80, 1.66) |  |  | 0.45 |
| Dressings | | Ref | 0.77 (0.55, 1.09) | 1.05 (0.75, 1.47) |  |  | 0.50 |
| Snacks | | Ref | 1.23 (0.87, 1.75) | 1.22 (0.87, 1.70) |  |  | 0.40 |
| **All-cause mortality** | |  |  |  |  |  |  |
| Total UPF | | Ref | 0.93 (0.72, 1.19) | 1.36 (1.06, 1.74) |  |  | <0.01 |
| Breakfast Foods | | Ref | 1.01 (0.79, 1.29) | 1.01 (0.79, 1.29) |  |  | 0.95 |
| Processed Meats | | Ref | 1.01 (0.78, 1.30) | 1.34 (1.05, 1.73) |  |  | <0.01 |
| Sweets | | Ref | 0.94 (0.73, 1.20) | 1.16 (0.91, 1.47) |  |  | 0.13 |
| Sugar-Sweetened Beverages | | Ref | 1.10 (0.86, 1.40) | 1.15 (0.90, 1.47) |  |  | 0.30 |
| Dairy | | Ref | 0.93 (0.73, 1.18) | 0.96 (0.76, 1.23) |  |  | 0.86 |
| Mixed Dishes | | Ref | 1.04 (0.81, 1.35) | 1.36 (1.05, 1.76) |  |  | 0.01 |
| Dressings | | Ref | 0.86 (0.68, 1.10) | 1.07 (0.84, 1.37) |  |  | 0.37 |
| Snacks | | Ref | 1.09 (0.85, 1.40) | 1.00 (0.78, 1.28) |  |  | 0.80 |
|  | **DAG defined minimal sufficient adjustment set**: age at diagnosis, baseline educational level (≤ high school graduate, some college, ≥ college graduate), health insurance status (Private, Medicare/Medicaid, Uninsured, Unknown), household income (<$15,000, $15,000-$29,999, ≥$30,000, Unknown), nSES (continuous), and marital status (married/living as married, widow/divorced/separated, single/never married). | | | | | | |

**Supplemental Table 5.** Association of pre-diagnosis UPFs consumption (serving/day vs. % of daily total energy intake) with breast cancer-specific mortality and all-cause mortality among the subgroup of participants with food composition information available (n=1298 from Women’s Circle of Health and Women’s Circle of Health Follow-Up Study)

|  | **UPFs** | **UPFs Consumption by tertiles** | | |
| --- | --- | --- | --- | --- |
|  | Continuous  (per 1 serving increase, or per 10% increase of UPF in %kcal/day) | Tertile1 | Tertile 2 | Tertile3 |
| **Breast cancer-specific mortality** |  |  |  |  |
| No. of events (n= 140) | 140 |  |  |  |
| UPF in servings/day, DAG^a^ defined model | 1.05 (1.00, 1.10) | Ref | 1.07 (0.66, 1.75) | 1.99 (1.20, 3.28) |
| UPF in servings/day, DAG defined model + total energy intake | 1.01 (0.92, 1.11) | Ref | 0.99 (0.60, 1.63) | 1.41 (0.72, 2.78) |
| UPF in servings/day, DAG defined model + nutrient density method for total energy intake adjustment | 1.02 (0.93, 1.13) | Ref | 0.99 (0.60, 1.63) | 1.31 (0.71, 2.82) |
| UPF in % of daily total energy intake, DAG defined model | 1.05 (0.91, 1.20) | Ref | 1.13 (0.70, 1.82) | 1.33 (0.75, 2.12) |
| UPF in % of daily total energy intake, DAG defined model + residual method for total energy intake adjustment | 1.03 (0.92, 1.15) | Ref | 0.72 (0.44, 1.19) | 1.04 (0.65, 1.68) |
| **All-cause mortality** |  |  |  |  |
| No. of events (n=256) | 256 |  |  |  |
| UPF in servings/day, DAG^a^ defined model | 1.07 (1.04, 1.11) | Ref | 1.09 (0.79, 1.51) | 1.65 (1.21, 2.24) |
| UPF in servings/day, DAG defined model + total energy intake | 1.04 (0.97. 1.11) | Ref | 1.00 (0.71, 1.39) | 1.24 (0.82, 1.88) |
| UPF in servings/day, DAG defined model + nutrient density method for total energy intake adjustment | 1.06 (0.96, 1.71) | Ref | 1.16 (0.74, 1.63) | 1.22 (0.81, 1.78) |
| UPF in % of daily total energy intake, DAG defined model | 1.08 (0.97, 1.19) | Ref | 1.20 (0.88, 1.64) | 1.30 (0.96, 1.77) |
| UPF in % of daily total energy intake, DAG defined model + residual method for total energy intake adjustment | 1.04 (0.96, 1.13) | Ref | 1.06 (0.78, 1.44) | 1.17 (0.86, 1.59) |

^a^ DAG defined minimal sufficient adjustment set): age at diagnosis (continuous, years), baseline educational level (≤ high school graduate, some college, ≥ college graduate), health insurance status (Private, Medicare/Medicaid, Uninsured, Unknown), household income (<$15,000, $15,000-$29,999, ≥$30,000, Unknown), nSES (continuous), and marital status (married/living as married, widow/divorced/separated, single/never married)


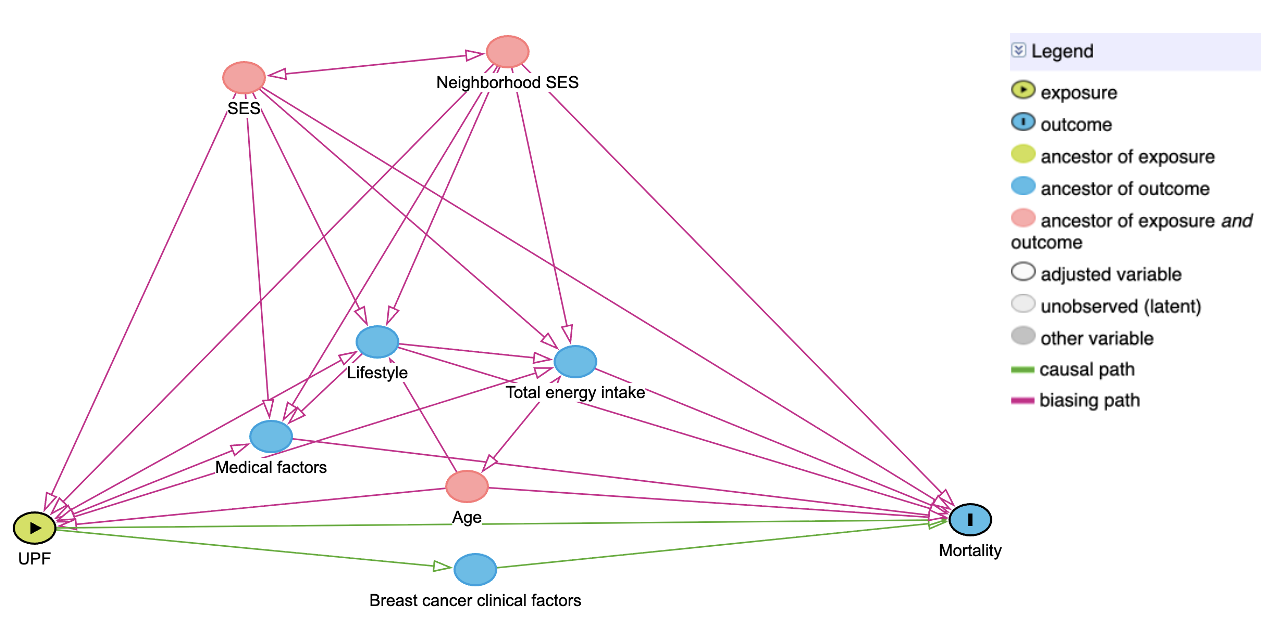


**Supplemental Figure 1**. DAG^22^ for the association of pre-diagnosis UPFs consumption with mortality among Black women with breast cancer in subgroups, the Women’s Circle of Health and Women’s Circle of Health Follow-Up Study (n=1,733)


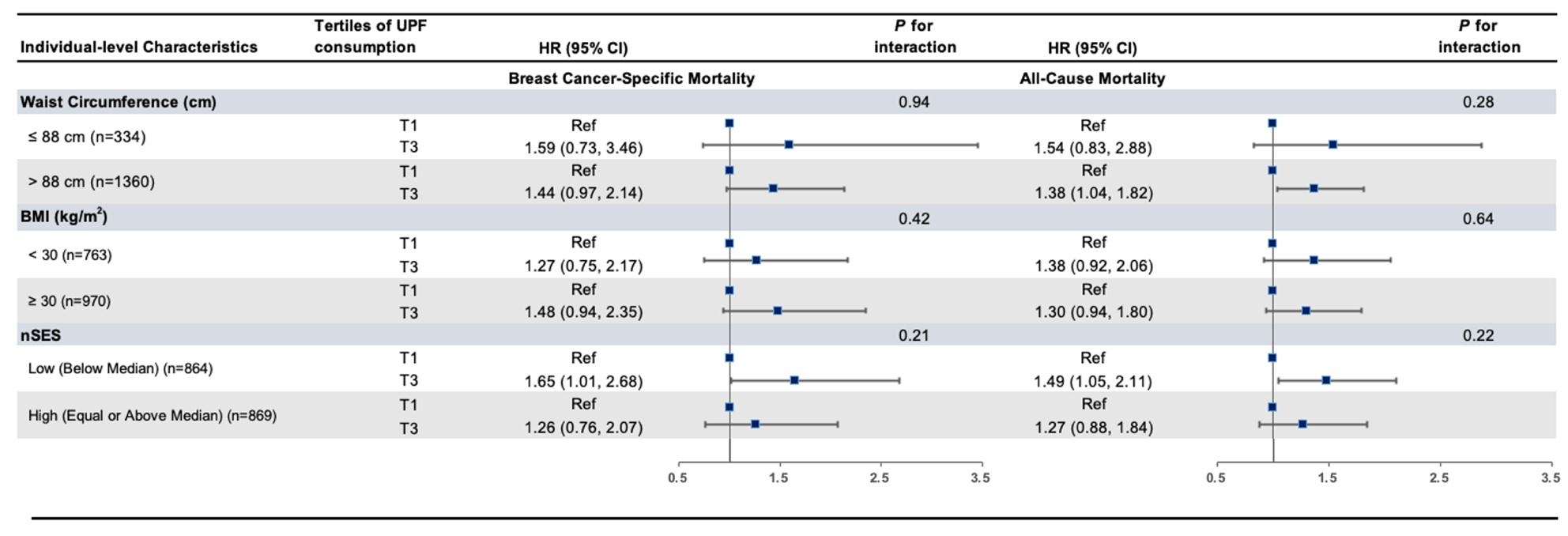


**Supplemental Figure 2**. Association of pre-diagnosis UPFs consumption with mortality among Black women with breast cancer in subgroups, the Women’s Circle of Health and Women’s Circle of Health Follow-Up Study (n=1,733)

Abbreviations: nSES: neighborhood socioeconomic status
